# Transoral Endoscopic Adenoidectomy

**DOI:** 10.1155/2009/949315

**Published:** 2009-07-28

**Authors:** Amr El-Badrawy, Mosaad Abdel-Aziz

**Affiliations:** Department of Otorhinolaryngology, Faculty of Medicine, Cairo University, Egypt

## Abstract

*Objective*. Adenoid curette guided by an indirect transoral mirror and a headlight is a simple and quick procedure that has already been in use for a long time, but this method carries a high risk of recurrence unless done by a well-experienced surgeon. The purpose of this paper was to evaluate the efficacy of transoral endoscopic adenoidectomy in relieving the obstructive nasal symptoms. *Methods*. 300 children underwent transoral endoscopic adenoidectomy using the classic adenoid curette and St Claire Thomson forceps with a 70^∘^ Hopkins 4-mm nasal endoscope introduced through the mouth and the view was projected on a monitor. Telephone questionnaire was used to follow-up the children for one year. Flexible nasopharyngoscopy was carried out for children with recurrent obstructive nasal symptoms to detect adenoid rehypertrophy. *Results*. No cases presented with postoperative complications. Only one case developed recurrent obstructive nasal symptoms due to adenoid regrowth and investigations showed that he had nasal allergy which may be the cause of recurrence. *Conclusion*. Transoral endoscopic adenoidectomy is the recent advancement of classic curettage adenoidectomy with direct vision of the nasopharynx that enables the surgeon to avoid injury of important structures as Eustachian tube orifices, and also it gives him the chance to completely remove the adenoidal tissues.

## 1. Introduction

Adenoidal hypertrophy is a common condition in children and can cause symptoms such as mouth breathing, nasal discharge, snoring, sleep apnea, and hyponasal speech [1] it also contributes to the pathogenesis of rhinosinusitis, recurrent otitis media, and otitis media with effusion [2].

Several adenoidectomy methods have been well described in the literature. Adenoid curette guided by an indirect transoral mirror and a headlight is a simple and quick procedure that has already been in use for a long time, but this method carries a high risk of recurrence unless done by a well-experienced surgeon [3, 4]. Recent methods, such as curved suction electrical coagulator [3] and the curved microdebrider shaver transorally [4, 5] guided by a transoral indirect mirror or a 45-degree endoscope, have successfully been used. Endoscopic-guided adenoidectomy using a classic adenoid curette has also been described [6]. Becker et al. [7] removed the adenoidal tissues transnasally combined with transorally under endoscopic visualization. Koltai et al. [8] described a power-assisted adenoidectomy technique without the use of nasal endoscope while Yanagisawa and Weaver [9] used the same technique under endoscopic vision but they found difficulty in maneuvering the microdebrider tip into the nasopharynx.

Each of these methods has its advantages and disadvantages; however, the symptoms of adenoid hypertrophy may recur or even persist after removal of the adenoid [10].

In this paper, we described an easy transoral endoscopic removal of adenoidal tissues aiming to assess the efficacy of this method in relieving the obstructive nasal symptoms.

## 2. Methods

Three-hundred patients were included in this study, all had adenoid (with or without tonsillar) hypertrophy. One hundred eighty seven were males and one hundred thirteen were females, their ages ranged between three to seven years with a mean age of four years and four months. All cases were collected from The Otolaryngology Clinics of Kasr El-Aini and Abu El-Rich Children Hospital of Cairo University in the period from January 2003 to December 2006. Informed consents were obtained from the parents of the patients, and the principles outlined in the Declaration of Helsinki were followed.

All patients were subjected to the following.

### 2.1. Preoperative Assessment

- General examination to exclude any other medical problems.
- Full ENT examination and history taking.
- Lateral X-ray nasopharyngeal radiographs.
- Tympanometry for cases suspected to have middle ear effusion.
- Routine lab investigations.

The following inclusion criteria were adopted.

- Adenoid is the only cause of nasal obstruction.
- No age group was excluded.
- No past history of cleft palate repair.
- Cases with submucous cleft palate were excluded.
- Cases with bleeding or coagulation defects were excluded.
- Cases missed followup were excluded.

### 2.2. Operative Technique

**Figure 1:**
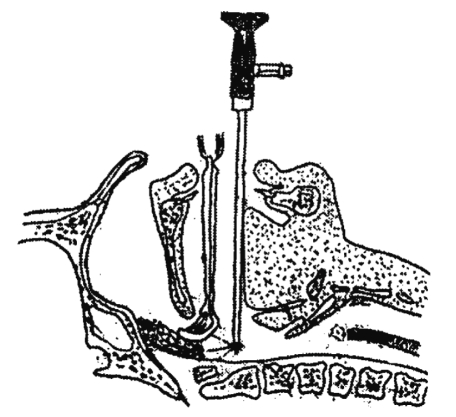
The adenoid curette inserted into the nasopharynx while the 70-degree nasal endoscope is introduced behind it.

Under general anesthesia with oral endotracheal intubation, a Boyle-Davis mouth gag was used to open the mouth. The soft palate was retracted with bilateral rubber catheters passed from the nose to the mouth and the two ends clamped tightly by artery forceps. The 70° Hopkins 4-mm nasal endoscope is introduced through the mouth, and the adenoid mass is identified (Figure 1). A camera mounted on the endoscope and the endoscopic view is projected on a monitor. Curettage of the main adenoid mass was carried out using adenoid curette, with removal of residual adenoidal tissues using St Claire Thomson forceps while suction is used to clear the field, the bleeding is usually minimal especially after complete removal of all nasopharyngeal lymphoid tissues. At the end of the procedure, a pack of gauze was inserted into the nasopharynx for few minute or till removal of the tonsils (if indicated). Tonsillectomy was carried out for seventy children and myringotomy with insertion of ventilation tubes was carried out for one-hundred and four cases. 

### 2.3. Postoperative Followup

Routine postoperative treatment in the form of antibiotics and analgesics for one week was given. The postoperative data were recorded for all cases. One year after the operation, the parents or caregivers of all children were contacted by telephone. They were asked to answer if the preoperative obstructive symptoms recurred or not. Those children with recurrent symptoms at the time of interview were invited to undergo flexible nasopharyngoscopy, and if they were found to have adenoid regrowth, we planned to search for the cause.

## 3. Results

The study sample (*n* = 300) had a mean age of 4 years and 4 months at the time of the adenoidectomy. No cases presented with postoperative complications such as hemorrhage, airway problems, Grisel's syndrome, stenosis, or velopharyngeal insufficiency. Telephone questionnaire showed recurrent obstructive symptoms in one case only; the child and his parents were invited for assessment. He was a male child aged 5 years and 3 months and he underwent adenoidectomy without tonsillectomy accompanied with myringotomy and ventilation tube insertion. His parents stated that the child started to snore 5 months after removal of his adenoid; the father gave a history of allergic rhinitis and bronchial asthma. Flexible nasopharyngoscopy for this patient showed recurrent obstructive adenoid, mainly in the right side of the nasopharynx. Otoscopic examination showed that both tubes were still in place without complications. Because of family history of allergic rhinitis, the child was subjected to skin prick test and he showed positive result with dust mite allergens (Dermatophagoides pteronyssinus and Dermatophagoides farinae; tests 708 and 725 manufactured by Allergopharma Co.). The child was subjected to revision transoral endoscopic adenoidectomy with antiallergic treatment; he showed no recurrent obstructive symptoms till the end of the study.

## 4. Discussion

Adenoidectomy is a safe and effective procedure, regardless of the method employed. Many different instruments and techniques have been utilized throughout the history of the procedure [11]. Removal of adenoid using transoral mirror is the most commonly used method; although the indirect visualization of adenoidal tissue, the incidence of recurrence is not known. In this study, we described an easy transoral endoscopic removal of adenoidal tissues using the adenoid curette and St Claire Thomson forceps. The advantage of this method is the direct visualization of the operative field which would decrease the incidence of residual lymphoid tissues, also it helps to avoid Eustachian tube injury that may causes its fibrosis. Moreover, this procedure dose not need expensive equipments as all used instruments are already present in the operating theatre, it may takes 2-3 minutes more than the conventional mirror method. However, transoral endoscopic removal may need some experience for junior surgeons that are usually unacquainted with the transoral view, moreover the wear and tear of the endoscope with each use may increase the expenses. Schaffer and Wong [12] stated that there is a short learning curve when applying endoscopic sinus techniques to the adenoidectomy procedure, but they believe otolaryngologists will find transoral endoscopic adenoidectomy to be easily learned and clinically successful, with little chance of adenoid regrowth. Wan et al. [6] reported that teaching the transoral endoscopic adenoid removal to trainee surgeons is much easier when combined with the video monitoring facilities.

Recurrent adenoids are most probably due to regrowth of residual lymphoid tissues left as a result of blind removal [2]. However, Buchinsky et al. [13] commented that adenoids rarely, if ever, regrow enough to cause symptoms of nasal obstruction after adenoidectomy that includes visualization and electrocautery of the adenoid bed.

Our study showed recurrent adenoid in 1 out of 300 cases (0.33%); Schaffer and Wong [12] used a similar technique for adenoid removal and they reported recurrence only in 2 patients out of 4000 adenoidectomies performed. However, many authors [12, 14] reported that recurrent adenoid after its removal may be attributed to nasal pathology, so we suggest that nasal allergy may play a role in recurrence in our case.

Cannon et al. [15] used the 4-mm 0 telescope transnasally at the end of the routine transoral adenoidectomy and they found residual adenoid tissue in all cases of their 236 except for 12 cases (5.1%); therefore they stressed that direct visualization is very important during the procedure.

Wan et al. [6] performed transoral curettage adenoidectomy guided with trans-nasal endoscope on 13 children, they reported marked improvement with no recurrence of obstructive symptoms. However, the sample of their patients was small and the procedure is very difficult as introduction of the curette into the nasopharynx may be accompanied with bleeding that obscures the view, while our method is much easier as the palate was retracted by 2 catheters to open the nasopharynx facilitating insertion of the curette above the superior border of the adenoid. In the technique of Wan et al. [6] we cannot retract the palate while the nasal endoscope is passing through the nose as the child's nose is very difficult to accommodate a catheter and an endoscope.

Kamel and Ishak [16] described trans-nasal endoscopic removal of the adenoid using the instruments of endoscopic sinus surgery, and they achieved improvement in 94% of their cases.

Partial transnasal endoscopic adenoidectomy has been described in cases that complained of obstructive adenoid with submucous cleft palate [17, 18]. The advantage of the endoscopic vision in these cases is to avoid complete adenoid removal that may results in velopharyngeal insufficiency.

None of our cases developed postoperative complications, as direct well vision would decrease haemorrage and if it happened it can be easily controlled. It also decreases the chance of injury of Eustachian tube orifices and violent removal of normal nasopharyngeal tissues that may result in fibrosis and stenosis.

This study was designed to evaluate the efficacy of transoral endoscopic removal of the adenoid, and it does not address the superiority of this method over other methods. To address this issue we must do randomized controlled trial to compare different methods of adenoid removal. However, we strongly advise randomized trials to compare this method with other methods regarding the operative time, the amount of intraoperative blood loss, the postoperative complications, and the incidence of recurrent symptoms.

In conclusion, transoral endoscopic adenoidectomy is the recent advancement of classic curettage adenoidectomy with good direct vision of the nasopharynx that enables the surgeon to avoid injury of important structures as Eustachian tube orifices and also gives him the chance to completely remove the adenoidal tissues.
